# Surgery for Pituitary Tumor Apoplexy Is Associated with Rapid Headache and Cranial Nerve Improvement

**DOI:** 10.3390/curroncol29070390

**Published:** 2022-07-12

**Authors:** Kevin A. Cross, Rupen Desai, Ananth Vellimana, Yupeng Liu, Keith Rich, Gregory Zipfel, Ralph Dacey, Michael Chicoine, Cristine Klatt-Cromwell, Jonathan McJunkin, Patrik Pipkorn, John S. Schneider, Julie Silverstein, Albert H. Kim

**Affiliations:** 1 Department of Neurological Surgery, Washington University School of Medicine, St. Louis, MO 63110, USA; cross.k@wustl.edu (K.A.C.); rupen.desai@wustl.edu (R.D.); vellimana@wustl.edu (A.V.); yupeng.liu@wustl.edu (Y.L.); richk@wustl.edu (K.R.); zipfelg@wustl.edu (G.Z.); daceyr@wustl.edu (R.D.); chicoinem@wustl.edu (M.C.); 2 The Brain Tumor Center, Siteman Cancer Center, Washington University School of Medicine, St. Louis, MO 63110, USA; 3 Department of Otolaryngology-Head and Neck Surgery, Washington University School of Medicine, St. Louis, MO 63110, USA; klatt-cromwell@wustl.edu (C.K.-C.); jonathan.mcjunkin@carle.com (J.M.); ppipkorn@wustl.edu (P.P.); jsschnei@wustl.edu (J.S.S.); 4 Division of Endocrinology, Metabolism, and Lipid Research, Washington University School of Medicine, St. Louis, MO 63110, USA; jsilverstein@wustl.edu

**Keywords:** pituitary tumor apoplexy, pituitary apoplexy, ophthalmoplegia, recovery, headache

## Abstract

Pituitary tumor apoplexy (PTA) classically comprises sudden-onset headache, loss of vision, ophthalmoparesis, and decreased consciousness. It typically results from hemorrhage and/or infarction within a pituitary adenoma. Presentation is heterologous, and optimal management is debated. The time course of recovery of cranial nerve deficits (CNDs) and headaches is not well established. In this study, a retrospective series of consecutive patients with PTA managed at a single academic institution over a 22-year period is presented. Headaches at the time of surgery were more severe in the early and subacute surgical cohort and improved significantly within 72 h postoperatively (*p* < 0.01). At one year, 90% of CNDs affecting cranial nerves (CNs) 3, 4, and 6 had recovered, with no differences between early (<4 d), subacute (4–14 d), and delayed (>14 d) time-to-surgery cohorts. Remarkably, half recovered within three days. In total, 56% of CN2 deficits recovered, with the early surgery cohort including more severe deficits and recovering at a lower rate (*p* = 0.01). No correlation of time-to-surgery and rapidity of recovery of CNDs was observed (*p* = 0.65, 0.72). Surgery for PTA is associated with rapid recovery of CNDs in the early, subacute, and delayed time frames, and with rapid headache improvement in the early and subacute time frames in 50% or more of patients.

## 1. Introduction

Pituitary tumor apoplexy (PTA) is a rare syndrome classically including sudden-onset headache, partial or complete loss of vision, ophthalmoparesis, and a decreased level of consciousness. It is thought to result from infarction and/or hemorrhage within a pituitary tumor. This causes the rapid expansion of sellar contents and the compression of adjacent structures, including optic nerves, optic chiasm, and intracavernous sinus cranial nerves (CNs) (e.g., 3/III, 4/IV, V1, V2, 6/VI), ultimately leading to cranial nerve deficits (CNDs) [1,2,3]. The pathogenesis of headache in PTA may involve pain fibers traveling within the sellar meninges and appears to correlate with increased intrasellar pressure (ISP) [4,5,6,7]. PTA was first described in a case series in 1950 of five patients who suffered severe symptoms, including blindness and death [8]. Subsequent studies proposed that early surgery to decompress the sella turcica and resect the tumor might improve patient outcomes and established a treatment paradigm for early surgery in this condition [9,10,11]. However, the definition of this condition has changed in the era of modern, high-resolution, and widely accessible magnetic resonance (MR) and computed tomography (CT) imaging, which can detect small and often subclinical hemorrhages within pituitary tumors [12,13,14,15]. With a wide spectrum of clinical severity evident in patients suffering from apparently similar pathophysiologic processes [16], controversy exists as to optimal treatment. For patients with visual symptoms (deficit of CN 2), most centers and guidelines continue to recommend prompt surgical decompression to preserve or improve visual function, although the timing of surgery is not well-defined [17]. The pituitary apoplexy score was proposed in 2011 to help guide management but does not include headache as a factor [18].

We performed a retrospective analysis of all patients who underwent surgery for PTA at a single institution in a 22-year period to ask if time-to-surgery correlates with more rapid resolution of headaches or CNDs.

## 2. Methods

### 2.1. Record Collection

Records of consecutive patients who underwent surgery for PTA at a single center between the years 2001 and 2022 were retrospectively reviewed. This study was approved by the Institutional Review Board of Washington University School of Medicine in St. Louis. Inclusion criteria included acute-onset headache in patients with radiologic evidence of hemorrhage or infarction within a pituitary tumor and pathology consistent with hemorrhage and/or necrosis within the adenoma. Patients without CNDs were also included. Details of the history of present illness, radiologic studies, surgical management, and follow-up were gathered via retrospective chart review. In six cases, the patient presented more than 60 days after ictus. For these patients, the duration of symptoms was censored at 60 days for subsequent analysis. Visual acuity, visual fields, and ocular motility data were obtained from combined records of ophthalmologists, neurosurgeons, and neurologists. Maximal tumor diameter was determined by preoperative imaging. MRI images were used when available, and CT images were used otherwise. Headache severity was obtained from nursing shift reports recorded at 0700 and 1900 daily in the electronic medical record and was marked categorically as “absent”, “mild,” “severe”, or “unable to obtain” based on the patient’s response. Headache data were gathered 72 h pre- and postoperatively prior to statistical analysis.

### 2.2. Statistical Analyses

Baseline characteristics of patients were compared using Pearson’s χ^2^ test or analysis of variance (ANOVA) for categorical or continuous variables, respectively. Pre- and postoperative headache scores were analyzed in two ways. Scores were first compiled and analyzed using Pearson’s χ^2^ test. Categorical headache responses were then converted to numerical scales using the following scores: Absent-1, Mild-2, and Severe-3. Scores were averaged in the pre- and postoperative period by each study subject and compared using the Wilcoxon matched pairs signed rank test. For CND improvement and resolution curves, Kaplan–Meier survival estimates were constructed. Survival curves were compared using the log-rank test, with significance set at *p* < 0.05. Cox proportional hazards regression analysis was used for multivariate analysis.

## 3. Results

### 3.1. Baseline Characteristics

Baseline characteristics of patients are presented in Table 1. In total, 59 patients were identified within the study period (40 male, 19 female). The median age at presentation was 54 years (a range of 17–94 years). The median follow-up time was 60 days (interquartile range 23–242 days). In total, 8 of 59 (13%) patients presented with a known history of pituitary neoplasm. 

The mean duration from symptom onset to surgery was 17 days, with a median of 11 days (interquartile range, 3–14 days). The early surgery cohort was defined as having undergone surgery <4 days after ictus, while the subacute and delayed cohorts underwent surgery between 4–14 days and >14 days, respectively. There was a higher proportion of male patients in the early and subacute treatment groups than in the delayed treatment group (*p* = 0.02). The mean maximal tumor diameter as measured by preoperative imaging was 2.5 cm within the entire study and was significantly greater in the early cohort (3.2 cm) than in the subacute (2.3 cm) and delayed cohorts (2.3 cm) (*p* = 0.02).

### 3.2. Headache Presentation and Resolution

**Figure 1:**
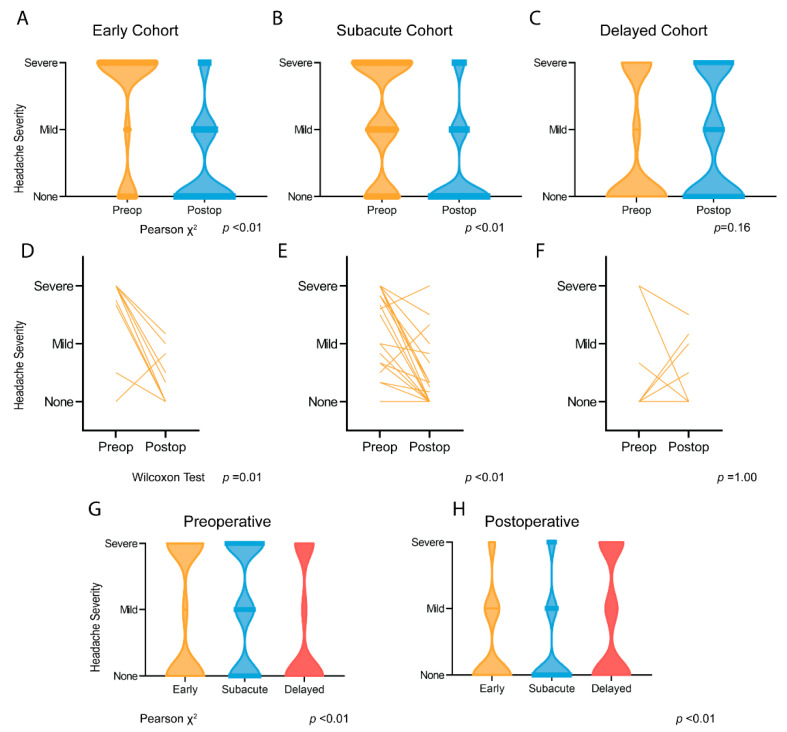
Pre- and Postoperative Headaches. Distributions of severity of headaches pre- and postoperatively in early (**A**), subacute (**B**), and delayed (**C**) cohorts. Individual subjects’ headache scores pre- and postoperatively in early (**D**), subacute (**E**), and delayed (**F**) cohorts. Distributions of headache severity by cohort in preoperative (**G**) and postoperative (**H**) time periods.

Headache data are displayed in Figure 1. All patients included in this study presented with headaches. Pre- and postoperative headache scores were available in 46 (77%) of 59 patients. In patients with available pre- and postoperative data, 11 (23%) headaches resolved before the 24 h period immediately preoperatively (three subacute, eight delayed). Within the early and subacute cohorts, the severity of headaches significantly decreased within 72 h following surgery (*p* < 0.01). Within the delayed surgical cohort, the distribution of headaches was not different between pre-and postoperative evaluation. Severe headaches in the immediate preoperative period were significantly more common in the early and subacute cohorts as compared to the delayed cohort (*p* <0.01). Among all postoperative patients, those within the delayed cohort reported severe headaches at a significantly higher rate (*p* < 0.01).

### 3.3. Clinical Presentation of Cranial Nerve Deficits

Baseline cranial nerve data are presented in Table 2. In total, 46 patients (78%) presented with at least one CN deficit. A total of 81 CNDs were detected, for an average of 1.3 per patient. Twenty-two (37%) patients presented with a CN2 deficit (7 unilateral, 15 bilateral), including partial or complete reduction in visual acuity or visual field. Five patients experienced total blindness in at least one eye by presentation (four bilateral, one unilateral). Therefore, 24% of all CN2 deficits were associated with complete blindness. The average time to presentation for patients with at least unilateral blindness was two days. Some of these patients experienced progressive visual loss to blindness over the course of many hours prior to presentation, which delayed their presentations. Three of five patients with at least unilateral blindness underwent surgery within 72 h of symptom onset. The remaining two patients fell into the subacute cohort. In total, 26 patients (44%) presented with a CN 3 deficit. Twelve patients (20%) presented with a CN 6 deficit. Only one CN 4 deficit was noted. Ten patients (16%) had deficits in both visual acuity and ocular motility.

### 3.4. Resolution and Improvement in Cranial Nerve Impairments

**Figure 2:**
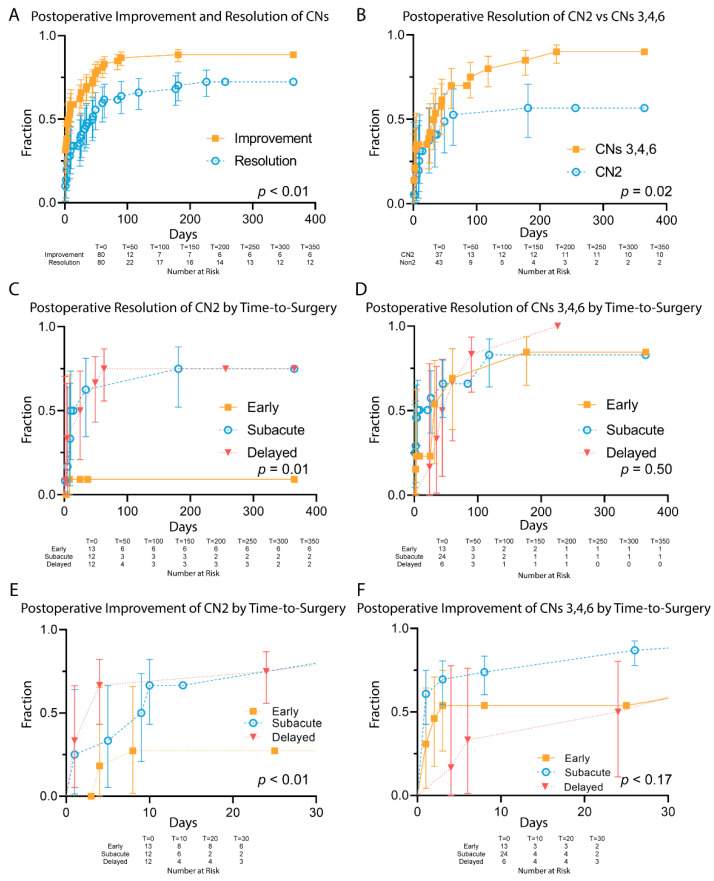
Postoperative Resolution and Improvement of Cranial Nerve Deficits. (**A**)—Postoperative Improvement and Resolution of CNDs. (**B**)—Resolution of CN2 vs CNs 3, 4, and 6. (**C**)—Resolution of CN2 by time-to-surgery cohort. (**D**)—Resolution of CNs 3, 4, and 6 by time-to-surgery cohort. (**E**)—Improvement in CN2 by time-to-surgery cohort. (**F**)—Improvement in CNs 3, 4, and 6 by time-to-surgery cohort.

Unlike with headaches, no CNDs were observed to resolve spontaneously prior to surgery within this study’s timeline. CN recoveries are displayed in Figure 2. At one-year follow-up, 72% of CNDs were completely resolved, with an additional 16% improved without full resolution. Deficits of CNs 3, 4, and 6 improved more frequently than visual deficits: 56% of CN 2 deficits resolved in one year versus 90% of CN 3, 4, and 6 deficits (*p* = 0.02). The severity of visual deficits was not evenly distributed among cohorts. Severe visual deficits in our series trended towards earlier surgical decompression (*p* = 0.06). By Cox Proportional Hazards multivariate analysis, this subset of patients was identified as having reduced rates of CN 2 resolution (*p* = 0.03). Among CN 2 deficits, 10% within the early surgery cohort resolved completely compared to 75% in each of the subacute and delayed cohorts (*p* = 0.01). Among early, subacute, and delayed cohorts, there were no significant differences in the resolution of CN oculomotor deficits at one year. 

**Figure 3:**
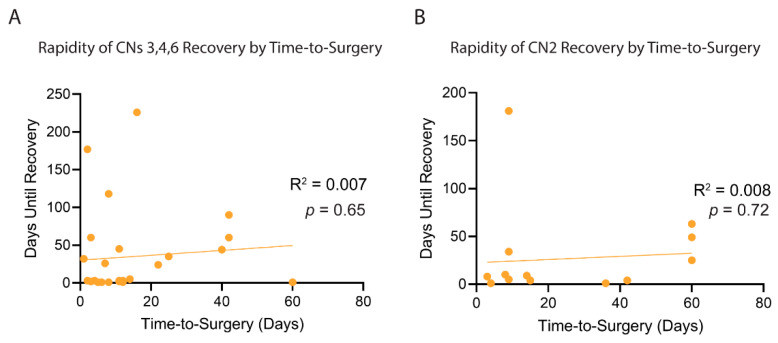
Correlation of Time to Surgery and Rapidity of Postoperative Recovery. Linear regression analysis and F-Test as performed to assess correlation in (**A**)—CNs 3, 4, and 6, and (**B**)—CN2.

In regression analysis of patients with CNDs that eventually recovered, time to surgery was not correlated with the rapidity of postoperative recovery for either CN2 or CNs 3, 4, and 6 (Figure 3). In total, 14 of 28 (50%) of CNs 3, 4, and 6 deficits recovered within the first three days postoperatively. Within seven days, 50% of CN2 and 55% of CNs 3, 4, and 6 deficits had resolved. Recovery within three days was observed in patients undergoing surgery 40 and 60 days after ictus.

### 3.5. Complications

Complications are visualized in Table 3 and included intraoperative postoperative DVT, postoperative diabetes insipidus (DI), postoperative heparin-induced thrombocytopenia (HIT), postoperative healthcare-associated pneumonia (HCAP), cerebrospinal fluid leak requiring the placement of a lumbar drain, and death during hospitalization. In the latter case, the patient was an 86-year-old woman whose death resulted from complications from HIT. There were no statistically significant differences between cohorts with regard to complications.

## 4. Discussion

The long-term neuro-ophthalmologic and endocrine outcomes of pituitary tumor apoplexy have been reviewed in the literature [19,20], but the time-course of recovery of CNDs and headache improvement has not yet been reported with this level of granularity, which is included in the analysis of time to surgery on outcomes.

Our observed rates of the long-term recovery of CNDs mirror the estimations of several previous series [21,22,23,24,25]. However, whereas some have reported improved visual recovery with early surgery [26,27,28], this association was not reflected in our data. It is possible that early surgery could have exacerbated vision loss in this cohort, but we propose instead that early surgical patients presented with more severe vision loss that resolved at a lower rate, as multivariate analysis confirmed blindness to be independently associated with persistent deficit. One-year outcomes were excellent for the recovery of CNDs of 3, 4, and 6, at 90%. Surgery was frequently associated with rapid recovery of these, with half of all CNDs affecting ocular movement resolving in the first 3 days after surgery. No difference in the rapidity of postoperative recoveries was detected between time-to-surgery cohorts, with several rapid CND recoveries also observed in the delayed treatment cohort. This raises the possibility that, in certain cases, surgery may expedite CND recovery even at delayed time points, though future prospective studies will be needed to test this hypothesis more rigorously.

PTA also causes debilitating headaches, the natural history and optimal treatment for which remain unclear. In a cohort of 44 patients treated with surgery, Zaidi et al. found headaches resolved at an average of 1.9 weeks postoperatively, and that all headaches resolved at the latest follow-up [29]. In the present study, we find patients who undergo surgery earlier have more severe headaches immediately before surgery, and that these are significantly improved within 72 h postoperatively. By contrast, most patients within the delayed surgical cohort (all of whom had previously experienced severe and sudden onset headaches) reported less severe, or absent, headaches by the time of surgery. Delayed surgical patients experienced no significant change overall in headache distribution after surgery. Hayashi and colleagues proposed that surgery could relieve headaches by reducing intrasellar pressure and showed improvement on a standardized headache score in PTA patients who underwent surgery. However, their postoperative assessment occurred three months after surgery. The data within the present study support that headache improvement can occur even within 72 h in the postoperative period.

One limitation of this study is its retrospective nature and inability to mitigate bias between sub-populations, though multivariate regression analysis helps to account for these. In future studies, propensity score matching could also potentially provide additional comparative strength between cohorts, though, in this study, discrepancies between calculated propensity scores of cohorts did not allow for close matching. This data set also reflects the practice of a single tertiary referral center and will be improved with a multi-institutional study. Finally, the headache data presented within are based on patients’ subjective responses to verbal nursing prompts. These data could potentially be improved with the use of a standardized tool such as the Visual Assessment Scale or Faces pain rating scale [30]. Despite these limitations, these data do provide useful insights into the postoperative recoveries of patients with PTA that could guide future investigation and practice.

## 5. Conclusions

The optimal management of PTA remains indeterminate. Previous studies have reported good endocrine and CN outcomes in subclinical PTA patients who underwent delayed surgery or medical management [31,32,33,34,35]. Furthermore, the definition of “early” and “delayed” surgery in this condition is debated. In this single-institution retrospective cohort study, the effects of surgical timing on headaches, vision, and extraocular movements were analyzed using <4 days and >14 days as cutoffs for “early” and “delayed”, respectively. This study includes a large cohort of patients that reflects the heterogeneity of this condition observed in modern neurosurgical practice. All patients experienced severe headaches and pituitary tumor hemorrhage/infarction, but many developed mild or no cranial nerve dysfunction. While long-term outcomes did not vary by time to surgery in PTA patients with minor visual deficits and/or deficits in ocular motility, surgery at all time points was associated with prompt improvement in cranial nerve deficits. Headaches improved significantly within 72 h in patients still experiencing severe headaches. The identification of the factors that might predict rapid headache or cranial nerve response to surgical decompression remains an important subject of future investigation.

## Figures and Tables

**Table 1:** Baseline Characteristics of Study Patients.

	Total	Operative Timeframe Cohort			*p* Value
		Early (<=72 h)	Subacute (4 d–14 d)	Delayed (>14d)	
Number of Patients (%)	59 (100)	13 (22)	27 (45)	19 (32)	-
Headaches	59 (100)	13 (100)	27 (100)	19 (100)	-
Radiologic Hemorrhage	59 (100)	13 (100)	27 (100)	19 (100)	-
Hemorrhage or Necrosis	59 (100)	13 (100)	27 (100)	19 (100)	-
Mean Time to Surgery (d)	17.6	1.9	9.0	40.4	<0.01
Mean Tumor Diameter (mm)	24.9	32.3	22.9	22.7	<0.01
Cranial Nerve Deficits	46 (78)	11 (85)	22 (81)	13 (68)	0.46
Blindness, Uni or Bilateral	5 (8)	3 (23)	2 (7)	0 (0)	0.06
Endoscopic Endonasal	56 (95)	12 (92)	25 (93)	19 (100)	0.47
Sublabial	3 (5)	1 (8)	2 (7)	0 (0)	

Preoperative characteristics of patients. Categorical variables were compared using Pearson’s χ^2^ test. Continuous variables were compared using ANOVA. Statistical significance set at *p* < 0.05.

**Table 2:** Cranial Nerve Deficits at Presentation.

**Cranial Nerve**	**Unilateral**	**Bilateral**	***p* Value**
II	7	15	
III	26	0	<0.01
VI	13	1	

In total, 46 patients (78%) presented with a cranial nerve deficit. CN2 deficits were frequently bilateral, while deficits affecting CNs 3, 4, and 6 were almost entirely unilateral (*p* < 0.01).

**Table 3:** Complications.

	Total	Operative Timeframe Cohort			*p* Value
		Early (<=72 h)	Subacute (4 d–14 d)	Delayed (>14 d)	
Deep Vein Thrombosis	2 (3)	0 (0)	2 (7)	0 (0)	0.29
Diabetes Insipidus	5 (8)	1 (7)	3 (11)	1 (5)	0.78
Heparin Induced Thrombocytopenia	1 (2)	1 (7)	0 (0)	0 (0)	0.17
Pneumonia	1 (2)	0 (0)	1 (3)	0 (0)	0.55
Cerebrospinal Fluid Leak	10 (17)	2 (15)	5 (19)	3 (16)	0.96
Death During Hospitalization	1 (2)	1 (7)	0 (0)	0 (0)	0.17

Pearson’s χ^2^ test was used to test for differences between cohorts.

## Data Availability

The data presented in this study are available on request from the corresponding author.
